# Can Physicochemical Properties Alter the Potency of Aeroallergens? Part 1 – Aeroallergen Protein Families

**DOI:** 10.1007/s11882-024-01172-8

**Published:** 2024-09-20

**Authors:** Carla S. S. Teixeira, Bruno Carriço-Sá, Caterina Villa, Isabel Mafra, Joana Costa

**Affiliations:** REQUIMTE-LAQV, Faculdade de Farmácia, Universidade do Porto, Rua de Jorge Viterbo Ferreira, 228, 4050-313 Porto, Portugal

**Keywords:** Aeroallergens, Physicochemical properties, Respiratory allergies, Protein allergenicity, Inhalant allergies, Allergic asthma, Allergic sinusitis

## Abstract

**Purpose of Review:**

Respiratory allergies are non-communicable diseases caused by the hypersensitivity of the immune system to environmental aeroallergens. The culprits are aero-transported proteins eliciting respiratory symptoms in sensitized/allergic individuals. This review intends to provide a holistic overview on the categorization of aeroallergens into protein families (Part 1) and to exploit the impact of physicochemical properties on inhalant protein allergenicity (Part 2). This first part will focus particularly on aeroallergen organization into families and how this classification fits their physicochemical properties.

**Recent Findings:**

Aeroallergen classification into protein families facilitates the identification of common physicochemical properties, thus aiding a better comprehension of known allergens, while predicting the behavior of novel ones. The available online databases gathering important features of aeroallergens are currently scarce.

**Summary:**

Information on distinct aeroallergen classification is still lacking, as data is dispersed and often outdated, hampering an efficient evaluation of new aeroallergens.

## Introduction

Respiratory allergies (RA) are conditions caused by the hypersensitivity of the immune system to certain allergens in the environment. They are known to be caused by shared sources of allergens and to induce similar clinical expression. However, they present some dissimilarities in their pathophysiologic mechanisms, being considered clinically distinct diseases. The most common types of RA include allergic rhinitis (hay fever), allergic asthma, allergic sinusitis, and occupational allergies. The prevalence of RA is influenced by factors such as geographical area, environmental exposure, and genetic predisposition, as well as by the condition typology. Allergic rhinitis is probably the most prevalent RA affecting globally approximately 10–30% of adults and 40% of children [1]. Allergic asthma is also estimated to have a major impact on 5–10% of the population, with higher prevalence in urban areas and industrialized countries [2, 3].

Allergic asthma is described as a chronic lung disease with multifactorial components that are associated with airway obstruction, mucus production, and bronchospasm [4]. Its clinical expression includes wheezing, coughing, shortness of breath, and chest tightness, being triggered by aeroallergens [5]. Allergic asthma is typically life-persistent, being a major non-communicable disease with a significant impact on the life quality of individuals of all ages, thus representing a key public health issue and a global economic burden [6].

Allergic rhinitis is characterized by an immunoglobulin E (IgE)-mediated type 1 hypersensitivity reaction to a wide variety of aeroallergens, leading to a cascade of immunological/biochemical events responsible for the clinical expression of the disease, namely sneezing, nasal congestion, itching, and rhinorrhea for more than one hour during two or more consecutive days [7]. Allergic rhinitis can be categorized based on symptoms’ duration and severity, ranging from intermittent to persistent and mild to life-threatening, respectively. It can co-occur with other diseases from the spectrum of allergies, namely allergic asthma, atopic dermatitis, and food allergy (FA), meaning that these health conditions might share some common mechanisms [8]. Additionally, allergic rhinitis is widely correlated with sinusitis, but the link between them is still unknown [9]. The term allergic sinusitis has been used to define a systemic inflammation process of the local nasal airway, nasal-associated lymphatic tissue, the bone marrow, and the sinuses [10]. This condition is caused by aeroallergens leading to facial pain, pressure, nasal congestion, postnasal drip, and a reduced sense of smell. Its diagnosis involves a combination of clinical evaluation, imaging studies (such as computed tomography scan), and allergy testing [11, 12].

Along with the previous three health conditions, occupational allergies are among the most reported work-related diseases, with the respiratory tract (occupational asthma/rhinitis) and skin (allergic contact dermatitis) being the classical target organs. So far, more than 400 occupational agents are classified as potential “respiratory sensitizers”, inducing immunological responses that can be IgE or non-IgE-mediated [13]. Their diagnosis is performed by evaluating the medical history, followed by other exams, such as positive methacholine challenge result or bronchodilator responsiveness, determination of IgE-mediated sensitization, and specific inhalation challenge tests [14]. Besides being important RA, occupational allergies can also be related to FA, leading to very complex clinical manifestations, and greatly affecting the quality of life of allergic patients.

So far, the best strategy to avoid RA relies on the efficient primary prevention, meaning that susceptible individuals should ideally avoid contact with any of the potential sensitizers. However, it is estimated that 10% of the general population and between 50–80% of RA patients are polysensitized to multiple aeroallergens, either by displaying (i) cross-reactivity (one IgE repertoire binds different allergens sharing common structural features) or (ii) co-sensitization (several IgE repertoires binding unrelated/not structurally similar allergens) [15, 16]. This evidence greatly hampers any attempt at efficient RA management.

The causative agents are normally proteins from plant/animal/fungal origins, namely pollens, dust mites, animal dander, insect feces, fungi, or certain foods (flour and grains, fish, and seafood), being common triggers to all types of RA (allergic rhinitis, allergic asthma, allergic sinusitis, and occupational allergies). Aeroallergens (or inhalant allergens) are proteins belonging to well-defined protein families, often sharing collective features among them. Nonetheless, it is unknown how the physicochemical properties affect inhalant protein allergenicity. Due to the clinical relevance and prevalence of RA, it is important to fill knowledge gaps on the importance of aeroallergen physicochemical properties. In this two-part review, it is intended to highlight the paramountcy of the organization of the aeroallergens into families (Part 1) and to evaluate how different physicochemical properties affect inhalant protein allergenicity (Part 2). This Part 1 review will be particularly focused on the categorization of the aeroallergens into families and in what manner this classification fits their physicochemical properties.

## Aeroallergen Families

Allergens are compiled in few databases, being the World Health Organization/International Union of Immunologic Societies (WHO/IUIS) allergen nomenclature, the Allergome, the AllergenOnline, and the COMPARE among the most popular [17–20]. The WHO/IUIS allergen nomenclature database is probably the most widely used and frequently updated [21]. It provides information on allergen nomenclature, isoforms, variants and epitopes, protein biochemical name, species, protein molecular weight (MW), and routes of exposure, being organized according to major taxonomical groups and respective orders [17]. It also includes the links to databases on protein sequences (UniProt) and respective encoding genes (GenBank) [22, 23]. The other databases (COMPARE and AllergenOnline) also contain most of the referred information [24, 25]. The classification/inclusion of allergens into protein families can only be found in a different database, the AllFam [26]. This database catalogues the allergens into protein families, linking them to the WHO/IUIS Allergen Nomenclature and AllergenOnline databases, as well as correlating with data from the Pfam database [27]. However, its major gaps regard the absence of updated information since 2017 [26].

Currently, the WHO/IUIS allergen nomenclature lists 495 aeroallergens [17, 18], being divided in 103 families [26]. From those, 37% are distributed into 9 superfamilies of proteins, namely EF-hand superfamily (28 aeroallergens), profilin superfamily (28 aeroallergens), calycin superfamily (27 aeroallergens), prolamin superfamily (24 aeroallergens), subtilisin-like serine protease (21 aeroallergens), expansin superfamily (16 aeroallergens), Ole e 1-like proteins (16 aeroallergens), pathogenesis-related (PR-10) proteins, also known as Bet v 1 family (12 aeroallergens), and tropomyosins (11 aeroallergens), which are often subdivided in relevant groups containing important aeroallergens.

In this review, besides considering the number and importance of the allergens, the criteria of inclusion as inhalant protein families also considered their relevance as primary sensitizers of RA and their relationship with FA. PR-10 proteins and the nonspecific lipid-transfer proteins (nsLTP) (prolamin superfamily) are cases of important primary sensitizers of respiratory and food allergies in Europe.

### EF-hand Family

The EF-hand family encompasses a variety of calcium-binding proteins disclosing a conserved motif with a helix-loop-helix secondary structure composed of a 12-residue calcium-binding loop flanked by 2 α-helices of 12 residues in length. EF-hand protein members tend to occur in pairs, meaning that most family members have two, four, or six calcium-binding motifs. This motif is crucial for eukaryotic cellular signaling, and the pairing enables communication, and positive cooperativity, reducing the Ca^2+^ signal needed to attain protein saturation [28]. The conformational effects of Ca^2+^-binding are diverse and depend on the function of the protein. EF-hand proteins display varying sensitivities to Ca^2+^, influenced by the intrinsic binding ability of the EF-hand and the degree of cooperativity in Ca^2+^-binding to paired EF-hands. Additionally, two factors can affect their ability to bind Ca^2+^, namely its selectivity towards Mg^2+^ (a divalent cation that is in a much higher cytoplasmic concentration) and its interaction with a protein target [28]. The biological functions of EF-hand family members encompass signaling and calcium buffering or transport processes.

This family is composed of different groups of proteins, some of them with allergenicity. The EF-hand superfamily ranks first position in terms of identified and listed aeroallergens, sharing this position with profilins [17]. Presently, 28 aeroallergens belonging to EF-hand family are divided into different subfamilies of proteins, namely polcalcins (17 allergens), troponin C (5 allergens), myosin light chain (MLC) (5 allergens), and sarcoplasmic calcium-binding proteins (SCBP) (1 allergen) [17]. Polcalcins are composed of aeroallergens from plant sources (e.g., Bet v 4 – European white birch), while troponin C, MLC, and SCBP groups contain mostly aeroallergens from animal sources (e.g., Der f 39 – American house dust mite, Bla g 8 – German cockroach, Bos d 3 – cow, respectively) (Table 1).

**Table 1:** Summary of physicochemical properties of selected aeroallergen families of proteins

	Protein family	Biological function	MW	Protein structure	PTM	Ligand/cofactor-binding	Clinical relevance	Identified allergens
EF-hand superfamily	Polcalcins	Remains unclear, suggested regulatory functions (potential regulation of intracellular Ca^2+^ levels during pollen germination)	8–9 kDa	Quaternary structure (monomers characterized by α-helices forming a typical all-α protein fold)	No identified PTM	Ca^2+^-binding	Minor allergens	Aln g 4 (*Alnus glutinosa*), Amb a 9 (*Ambrosia artemisiifolia*), Amb a 10 (*Ambrosia artemisiifolia*), Art si 5 (*Artemisia sieversiana*), Art v 5 (*Artemisia vulgaris*), Bet v 3 (*Betula verrucosa*), Bet v 4 (*Betula verrucosa*), Bra r 5 (*Brassica rapa*), Che a 3 (*Chenopodium album*), Cyn d 7 (*Cynodon dactylon*), Jun o 4 (*Juniperus oxycedrus*), Sal k 7 (*Kali turgidum*), Ole e 3 (*Olea europaea*), Ole e 8 (*Olea europaea*), Par j 4 (*Parietaria judaica*), Phl p 7 (*Phleum pratense*), Syr v 3 (*Syringa vulgaris*)
	Troponin C	Regulatory/structural function (part of troponin complex together with troponin I/troponin T)	17–21 kDa	Tertiary structure (2 structurally homologous lobes, mainly composed of α-helices and loops long uncovered 9-turn helical linker)	No identified PTM	Ca^2+^-binding	Minor allergens	Bla g 6 (*Blattella germanica*), Der f 39 (*Dermatophagoides farinae*), Der p 39 (*Dermatophagoides pteronyssinus*), Per a 6 (*Periplaneta americana*), Tyr p 34 (*Tyrophagus putrescentiae*)
	MLC	Regulatory/structural function (multi-subunit complex with 2 heavy chains + 4 Ca^2+^-binding light chains)	18–28 kDa	Similar to troponin C, exists as a complex with myosin-heavy chains	No identified PTM	Ca^2+^-binding	Potentially minor allergens, although Blo t 26 is a major allergen	Bla g 8 (*Blattella germanica*), Blo t 26 (*Blomia tropicalis*), Der f 26 (*Dermatophagoides farinae*), Der p 26 (*Dermatophagoides pteronyssinus*), Per a 8 (*Periplaneta americana*)
	SCBP	Regulatory function	20–25 kDa	Tertiary structure (compact and globular conformation with hydrophobic core)	No identified PTM	Ca^2+^-binding	Minor allergens	Aed a 5 (*Aedes aegypti*)
-	Profilins	Regulatory function (key role of actin polymerization in the cytoskeleton, participation in cellular processes: motility, metabolism, endocytosis, signal transduction, gene transcription)	12–15 kDa	Quaternary structure (monomer, although with evidence of dimers or tetramers, composed by central 6-stranded antiparallel β-sheet and 2 α-helices, 1 at the N-terminal side perpendicular to the sheet and another parallel at the carboxy-terminal side)	Potentially phosphorylated in monomeric forms, not on dimers or oligomers	Bind to poly(L-proline), interaction with membrane-located phospholipids	Minor allergens	Aca f 2 (*Acacia farnesiana*), Ama r 2 (*Amaranthus retroflexus*), Amb a 8 (Ambrosia artemisiifolia), Amb t 8 (*Ambrosia trífida*), Art an 4 (*Artemisia annua*), Art v 4 (*Artemisia vulgaris*), Beta v 2 (*Beta vulgaris*), Bet v 2 (*Betula verrucosa*), Can s 2 (*Cannabis sativa*), Che a 2 (*Chenopodium album*), Cro s 2 (*Crocus sativus*), Cyn d 12 (*Cynodon dactylon*), Hel a 2 (*Helianthus annuus*), Sal k 4 (*Kali turgidum*), Koc s 2 (*Kochia scoparia*), Lig v 2 (*Ligustrum vulgare*), Mer a 1 (*Mercurialis annua*), Ole e 2 (*Olea europaea*), Ory s 12 (*Oryza sativa*), Par j 3 (*Parietaria judaica*), Phl p 12 (*Phleum pratense*), Pla l 2 (*Plantgo Lanceolata*), Pla a 4 (*Platanus acerifolia*), Pop n 2 (*Populus nigra*), Pro j 2 (*Prosopis juliflora*), Que ac 2 (*Quercus acutíssima*), Tyr p 36 (*Tyrophagus putrescentiae*), Zea m 12 (*Zea mays*)
Calycin superfamily	Lipocalins	Regulatory (metabolic, develop, and immunological processes) and transport functions (small hydrophobic molecules)	17–25 kDa	Quaternary structure (oligomers composed of eight-stranded anti-parallel β-barrel motif forming a ligand-binding pocket, and an α-helix)	N- and/or O-glycosylated or non-glycosylated	Bind to lipids, odorants, steroids, pheromones, retinoids, vitamins	Most lipocalins are major allergens	Bla g 4 (*Blattella germanica*), Bos d 2 (*Bos taurus*), Can f 1, Can f 2, Can f 4, Can f 6 *(Canis familiaris*), Cav p 1, Cav p 2, Cav p 3, Cav p 6 (*Cavia porcellus*), Equ c 1, Equ c 2 (*Equus caballus*), Fel d 4, Fel d 7 (*Felis domesticus*), Mes a 1 (*Mesocricetus auratus*), Mus m 1 (*Mus musculus*), Ory c 1, Ory c 2, Ory c 4 (*Oryctolagus cuniculus*), Per a 4 (*Periplaneta americana*), Rat n 1 (*Rattus norvegicus*)
	FABP	Transport function (intracellular transporters of hydrophobic metabolic intermediates and carriers of lipids between membranes)	12–18 kDa	Quaternary structure (monomers with tendency to self-associated (α-β structure: classic β-barrel with 10 antiparallel β-strands, covered with 2 α-helices at one end, creating an internal cavity))	No PTM identified	Fatty acids, retinol, retinoic acid, bile salts, and pigments	Most likely minor allergens	Aca s 13 (*Acarus siro*), Blo t 13 (*Blomia tropicalis*), Der f 13 (*Dermatophagoides farinae*), Der p 13 (Dermatophagoides pteronyssinus), Lep d 13 (*Lepidoglyphus destructor*), Tyr p 13 (*Tyrophagus putrescentiae*)
Prolamin superfamily	nsLTP	Transport function (intervene in the modulation of lipid composition, lipid transfer, vesicular trafficking, and signal transduction)	9.5–10.5 kDa	Tertiary structure (monomer composed of 4 α‐helices, stabilized by 4 highly conserved disulfide bridges, and connected by flexible loops, forming an internal, tunnel‐like, hydrophobic cavity)	No PTM identified	Fatty acids, fatty acyl-CoA, phospholipids, glycolipids, and cutin monomers	Potentially major allergens	Amb a 6 (*Ambrosia artemisiifolia*), Art v 3 (*Artemisia vulgaris*), Art an 3 (*Artemisia annua*), Art ar 3 (*Artemisia argyi*), Art ca 3 (*Artemisia capillaris*), Art gm 3 (*Artemisia gmelinii*), Art la 3 (*Artemisia lavandulifolia*), Art si 3 (*Artemisia sieversiana*), Can s 3 (*Cannabis sativa*), Ole e 7 (*Olea europaea*), Par j 1, Par j 2 (*Parietaria judaica*), Par o 3 (*Parietaria officinalis*), Pla or 3 (*Platanus orientalis*), Tri a 14, Tri a 44 (*Triticum aestivum*)
	α-amylases	Enzymatic function. Catalysis of α-1,4-glucosidic bonds in starch and related α-glucans with retention of α-anomeric configuration in the products	52–60 kDa	Tertiary structure. Complex monomer (polypeptide chain folded into 3 domains (A, B and C); characteristic (β/α)8-barrel with conserved catalytic core (domain A), a protrusion between third strand and third helix of (β/α)8-barrel having irregular β-like structure (domain B) and a C-terminal end of the amino acid sequence with key motif (domain C))	Some isoforms can suffer PTM	Ca^2+^-binding	Potentially major allergens	Asp o 21 (*Aspergillus oryzae*), Bla g 11 (*Blattella germanica*), Blo t 4 (*Blomia tropicalis*), Der f 4 (*Dermatophagoides farinae*), Der p 4 (*Dermatophagoides pteronyssinus*), Eur m 4 (*Euroglyphus maynei*), Per a 11 (*Periplaneta americana*), Tyr p 4 (*Tyrophagus putrescentiae*)
-	Subtilisin-like serine proteases	Enzymatic function (production of nutrients through protein cleavage and defense systems in immune evasion)	34–58 kDa	Quaternary structure. Normally monomers, but homodimerization can also occur (unique structural fold, with a highly twisted seven-stranded β-sheet flanked by 2 layers of α-helices)	Glycosylation	Ca^2+^-binding	Major allergens	Alt a 15 (*Alternaria alternata*), Asp fl 13 (*Aspergillus flavus*), Asp f 13, Asp f 18 (*Aspergillus fumigatus*), Asp n 18 (*Aspergillus niger*), Asp o 13 (*Aspergillus oryzae*), Asp v 13 (*Aspergillus versicolor*), Cla c 9 (*Cladosporium cladosporioides*), Cla h 9 (*Cladosporium herbarum*), Cur l 1, Cur l 4 (*Curvularia lunata*), Epi p 1 (*Epicoccum purpurascens*), Fus p 9 (*Fusarium proliferatum*), Pen b 13 (*Penicillium brevicompactum*), Pen ch 13, Pen ch 18 (*Penicillium chrysogenum*), Pen c 13 (*Penicillium citrinum*), Pen o 18 (*Penicillium oxalicum*), Per a 10 (*Periplaneta americana*), Rho m 2 (*Rhodotorula mucilaginosa*), Tri a 39 (*Triticum aestivum*)
-	Expansins	Regulatory function (participating in cell wall loosening processes, thereby aiding in the pollination process)	25–25 kDa	Tertiary structure. Monomer (2 domains: (i) N-terminal domain 1 (like the catalytic domain of family (GH45)) and (ii) domain 2 entailing β-sheets)	Glycosylation	Carbohydrates	Major allergens	Ant o 1 (*Anthoxanthum odoratum*), Cyn d 1 (*Cynodon dactylon*), Dac g 1 (*Dactylis glomerata*), Hol l 1 (*Holcus lanatus*), Lol p 1 (*Lolium perenne*), Ory s 1 (*Oryza sativa*), Pas n 1 (*Paspalum notatum*), Pha a 1 (*Phalaris aquatica*), Phl p 1 (*Phleum pratense*), Poa p 1 (*Poa pratensis*), Sor h 1 (*Sorghum halepense*), Uro m 1 (*Urochloa mutica*), Zea m 1 (*Zea mays*), Zoy m 1 (*Zoysia matrella*), Phl p 3 (*Phleum pratense*), Sor h 2 (*Sorghum halepense*)
-	Ole e 1-like proteins	Unknown function, although related to plant fertilization (probably regulation function)	15–20 kDa	Quaternary structure. Dimer or oligomer, depending on allergen (7-strand β-barrel fold with 3 conserved disulfide bonds, [EQT]-G-x-V–Y-C-D-[TNP]-C-R consensus pattern)	Partial N-glycosylation	Not reported	Potentially minor allergens	Aca f 1 (*Acacia farnesiana*), Ama r 1 (*Amaranthus retroflexus*), Asp f 35 (*Aspergillus fumigatus*), Beta v 1 (*Beta vulgaris*), Che a 1 (*Chenopodium album*), Cro s 1 (*Crocus sativus*), Fra e 1 (*Fraxinus excelsior*), Sal k 5 (*Kali turgidum*), Koc s 1 (*Kochia scoparia*), Lig v 1 (*Ligustrum vulgare*), Lol p 11 (*Lolium perenne*), Ole e 1 (*Olea europaea*), Phl p 11 (*Phleum pratense*), Pla l 1 (*Plantago lanceolata*), Pro j 1 (*Prosopis juliflora*), Syr v 1 (*Syringa vulgaris*)
-	PR-10 proteins (Bet v 1 family)	Defense function (against biotic/abiotic stresses) and transport function (transport of amphiphilic compounds)	15–17 kDa	Quaternary structure. Monomeric and oligomeric forms (contain seven-stranded antiparallel β-sheet with 25-amino acid α-helix (α3) at the C-terminus and 2 short α-helices (α1 and α2) positioned between β1 and β2 strands, forming a hydrophobic core)	Not reported	Fatty acids, cytokines, flavonoids, and sterols	Major allergens	Aln g 1 (*Alnus glutinosa*), Bet v 1 (*Betula verrucosa*), Can s 5 (*Cannabis sativa*), Car b 1 (*Carpinus betulus*), Cas s 1 (Castanea sativa), Cor a 1 (*Corylus avellana*), Fag s 1 (*Fagus sylvatica*), Ost c 1 (*Ostrya carpinifolia*), Que ac 1 (*Quercus acutissima*), Que a 1 (*Quercus alba*), Que i 1 (*Quercus ilex*), Que m 1 (*Quercus mongolica*)
-	Tropomyosin	Structural/regulatory function	34–38 kDa	Quaternary structure. Coiled-coil homo- or heterodimeric structures (composed of 2 parallel α-helices and 2 sets of 7 alternating actin-binding sites)	No PTM identified in aeroallergens, but in food allergens, tropomyosins can be glycosylated or acetylated	Not report	Classification of minor or major depending on the allergen	Aed a 10 (*Aedes aegypti*), Bla g 7 (*Blattella germanica*), Blo t 10 (*Blomia tropicalis*), Cho a 10 (*Chortoglyphus arcuatus*), Copt f 7 (*Coptotermes formosanus*), Der f 10 (*Dermatophagoides farinae*), Der p 10 (*Dermatophagoides pteronyssinus*), Lep d 10 (*Lepidoglyphus destructor*), Lep s 1 (*Lepisma saccharina*), Per a 7 (*Periplaneta americana*), Tyr p 10 (*Tyrophagus putrescentiae*)

*FABP*  fatty acid-binding protein, *MLC* myosin light chain, *nsLTP* nonspecific lipid transfer protein, *PTM* post-translational modification, *SCBP* sarcoplasmic calcium-binding protein

#### Polcalcins

**Fig. 1:**
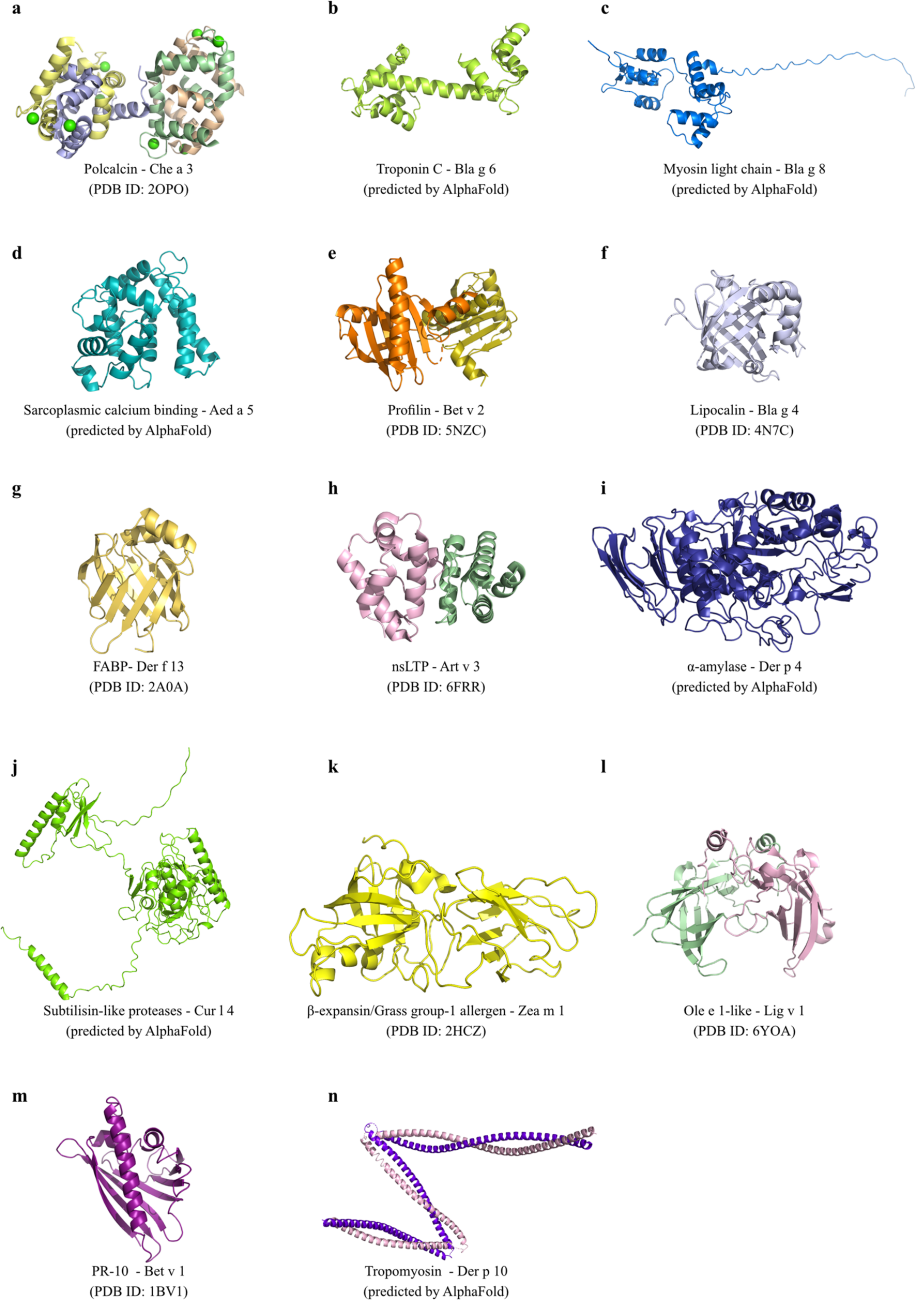
Pymol representation of: **a**) Crystal structure of polcalcin allergen Che a 3 from *Chenopodium album* with calcium ions represented as green spheres (PDB ID: 2OPO); **b**) 3D structure of troponin C allergen Bla g 6 from *Blattella germanica* predicted by AlphaFold; **c**) 3D structure of myosin light chain allergen Bla g 8 from *Blattella germanica* predicted by AlphaFold; **d**) 3D structure of sarcoplasmic calcium binding allergen Aed a 5 from *Aedes aegypdi* predicted by AlphaFold; **e**) Crystal structure of profilin allergen Bet v 2 from *Betula pendula* (PDB ID: 5NZC); **f**) Crystal structure of lipocalin allergen Bla g 4 from *Blattella germanica* (PDB ID: 4N7C); **g**) Crystal structure of FABP allergen Der f 13 from *Dermatophagoides farina* (PDB ID: 2A0A); **h**) Crystal structure of nsLTP allergen Art v 3 from *Artemisia vulgaris* (PDB ID: 6FRR); **i**) 3D structure of α -amylase allergen Der p 4 from *Dermatophagoides pteronyssinus* predicted by AlphaFold; **j**) 3D structure of subtilisin-like protease allergen Cur l 4 from *Cochliobolus lunatus* predicted by AlphaFold; **k**) Crystal structure of β -expansin/Grass group-1 allergen Zea m 1 from *Zea mays* (PDB ID: 2HCZ); **l**) Crystal structure of Ole e 1-like allergen Lig v 1 from *Ligustrum vulgare* (PDB ID: 6YOA); **m**) Crystal structure of PR-10 allergen Bet v 1 from *Betula pendula* (PDB ID: 1BV1); **n**) 3D structure of tropomyosin allergen Der p 10 from *Dermatophagoides pteronyssinus* predicted by AlphaFold). All protein structures are represented in cartoon and colored by chain

Based on the number of calcium-binding EF-hand motifs, polcalcins are categorized into three types, possessing different number of EF-hand motifs: two (e.g., Che a 3, white goosefoot), three (e.g., Bet v 3, European white birch), or four (e.g., Ole e 8, olive tree). The three-dimensional (3D) structure of polcalcins is characterized by α-helices forming a typical all-α protein fold (Fig. 1a, Table 1). The monomer, with a MW of 8–9 kDa, exhibits the characteristic polcalcin structural domain [29], but whose biological function remains unclear. However, given their specific localization in pollen and their calcium-binding ability, it is suggested that polcalcins may regulate intracellular calcium levels during pollen germination. Notably, the calcium-binding property of polcalcins influences their IgE-reactivity and thermostability [29]. Due to their high sequence identity and structural homology, these proteins are classified as panallergens [30]. Polcalcins are highly cross-reactive calcium-binding allergens specifically expressed in pollen tissues, meaning that sensitization to polcalcins is not associated with FA [29]. Less than 10% of allergic individuals are sensitized to polcalcins, so these proteins are classified as minor allergens [31]. The clinical expression of sensitization to polcalcins is still not clear, although symptoms are mostly described as mild. Patients sensitized to polcalcins often exhibit high cross-reactivity, as well as distinct IgE repertoires to different allergens, which highlights the need for monitoring these patients as they are susceptible of developing multiple pollen sensitizations [29]. So far, 17 polcalcins have been identified as aeroallergens in pollen (e.g. Ole e 3, olive) [17].

#### Troponin C

Troponin C is a calcium-binding protein that makes part of the troponin complex encompassing another 2 proteins, namely inhibitory (troponin I), and tropomyosin binding (troponin T). Troponin C is a muscle protein that connects the thin filaments and signals muscle contraction after binding Ca^2+^, presenting 2 structurally homologous lobes, mainly composed of α-helices and loops connected by a long uncovered 9-turn helical linker [32]. This protein plays a regulatory function, being common to animal sources. Sensitization to troponin C is often accompanied by cross-reactivity to its counterparts in foods, leading to an increased risk for individuals sensitized to this set of proteins [32]. So far, 5 troponin C have been classified as aeroallergens of insects/mites (e.g., Bla g 6, German cockroach) (Fig. 1b, Table 1), being classified as minor allergens [33].

#### Myosin Light Chain

MLC comprises a multi-subunit complex of two heavy chains and four calcium-binding light chains. It serves as a fundamental contractile protein present in animal cells. MLC from invertebrates, including cockroaches and crustaceans, can bind IgE, being classified as aeroallergens and food allergens, respectively. So far, 5 MLC have been identified as aeroallergens in insects/mites (e.g., Bla g 8, German cockroach) (Fig. 1c, Table 1).

#### Sarcoplasmic Calcium-binding Protein

SCBP are classified as invertebrate muscle proteins exhibiting a calcium buffering function, being commonly considered as food allergens, especially in crustaceans. SCBP structure is greatly compact and globular with a hydrophobic core, contrarily to the dumbbell-shaped structure of troponin C [34]. So far, only one aeroallergen (Aed a 5) was identified in the yellow fever mosquito (Fig. 1d, Table 1). The clinical expression of this aeroallergen is still unknown, although it is considered a minor allergen [35].

### Profilin Superfamily

Profilins are a family of actin-binding proteins composed of 125–153 amino acids, with MW of 12–15 kDa and isoelectric points (PI) between 4.3–9.2 [36]. They have been characterized as monomers, although dimers and tetramers have also been reported for profilin structures [37]. These proteins are key regulators of actin polymerization in the cytoskeleton, participating in several cellular processes, including motility, metabolism, endocytosis, signal transduction, and gene transcription [38]. Profilins are ubiquitous in most eucaryotic cells, exhibiting high sequence identity (> 70%), and structural homology, even among distantly related family members, rendering their classification as panallergens [29] and presenting high levels of cross-reactivity among plant-allergenic sources (Table 1). However, the high structural homology between plant and mite profilins might not result in clinically relevant respiratory allergies, as there is no evidence of cross-reactivity between these 2 groups [39]. Bet v 2 (European white birch) pollen (Fig. 1e) was the first allergenic profilin to be identified in 1991 and since then, other allergenic profilins have been discovered in pollen, latex, and plant foods. Bet v 2 is constituted by a central 6-stranded antiparallel β-sheet and two helices, one at the amino-terminal side perpendicular to the sheet and another parallel at the C-terminal side.

Presently, profilins share the first place with EF-hand family in terms of the number of identified molecules, accounting for 28 profilins classified as aeroallergens [17]. Allergenic inhalant profilins are mainly found in plant pollens (e.g., Cro s 2, saffron) and mites (e.g., Tyr p 36, storage mite), being important triggers of RA (e.g., allergic asthma) and relevant primary sensitizers of FA. Profilins have been recognized as clinically relevant aeroallergens, with sensitized/allergic subjects clinically reacting to multiple allergen sources [40]. Typically, profilins are associated with mild respiratory symptoms, but recent data seem to highlight their importance as causative agents for severe/systemic allergic reactions like anaphylaxis, especially in FA [40]. In Europe, 20–30% of individuals allergic to pollen are IgE-reactive to profilins [41, 42], leading to a minor allergen classification. However, there is no correlation between the level of IgE-reactivity of individual patients to specific profilins and their sensitizing source, signifying that sensitization could be linked to any aeroallergenic profilin. This fact highlights the usefulness of profilins as a common marker for polysensitization for diagnosis and therapeutic approaches [43].

### Calycin Protein Superfamily

The calycin superfamily is widely dispersed throughout all kingdoms and gathers a miscellaneous group of proteins known for their ability to bind small hydrophobic molecules. These proteins are involved in a variety of biological processes, including transport and storage of lipids, regulation of gene expression, and immune responses. The calycin core fold encompasses an eight-stranded calyx-shaped antiparallel β-barrel, which forms a hydrophobic binding cavity [44]. This calycin superfamily is composed of different families of allergenic proteins, namely lipocalins and fatty acid-binding proteins (FABP), ranking the second position in terms of the total number of identified aeroallergens (27 proteins) (Table 1) [44].

#### Lipocalins

Lipocalins are a diverse group of proteins with multiple biological roles that include (i) the transport of small hydrophobic molecules and (ii) the regulation of various metabolic, development, and immunological processes [45]. Lipocalins present 150–250 residues and a MW of 17–25 kDa, being extracellularly located. Although the sequence identity among lipocalins is generally low (20–30%), it can be higher than 50% in some cases, and with well-preserved 3D structures, sharing an eight-stranded antiparallel β-barrel motif that forms a ligand-binding pocket, and an α-helix (Table 1). Allergenic lipocalins sensitize efficiently, with an average sensitization rate > 50%. Presently, 21 lipocalins are classified as aeroallergens, including allergens like Bla g 4 (cockroaches) (Fig. 1f), Mus m 1 (mouse urine), and Can f 1 and Can f 2 (dog), most of them behaving as major allergens. Lipocalins are predominantly abundant in mucosa and skin epithelia, being extensively observed in body fluids and secretions, which makes them prevalent in indoor aeroallergens [17].

#### Fatty Acid-Binding Proteins

Cytosolic (cytoplasmic) fatty acid-binding proteins (FABP) are a family of proteins primarily located in the cytosol, playing a crucial role in lipid metabolism by acting as intracellular transporters of hydrophobic metabolic intermediates and as carriers of lipids between membranes. FABP exhibit significant sequence and structural similarity, being small soluble proteins with 12–18 kDa and 110–160 residues. They are typically monomers but with tendency to form self-associated structures [46]. These proteins have a mixed α-β structure, with a classic β-barrel made up of 10 antiparallel β-strands and covered with a pair of α-helices at one end, creating an internal cavity that serves as the ligand-binding site [47, 48]. Ligand specificity and affinity are influenced by the side chains of amino acids that extend into the cavity, particularly polar residues that engage in hydrogen bonding and electrostatic interactions with the polar head groups of the ligands. For instance, arginine binds fatty acids, while glutamine binds retinoids. The antiparallel β-barrel fold is also common to lipocalins, which similarly bind small hydrophobic molecules. However, sequence similarity between FABP and lipocalins is limited to a short N-terminal motif. Presently, 6 FABP are identified as aeroallergens, all belonging to mites (e.g., Der f 13, American house dust mite (HDM)) (Fig. 1g, Table 1) [17].

### Prolamin Superfamily

Proteins of the prolamin superfamily are composed of a high content of glutamine and proline residues, which is the typical trace of prolamins. Members of this superfamily show little sequence homology, although preserving a common cysteine skeleton and α-helical structures [49]. They have a preserved pattern of eight cysteine residues, being responsible for their 3D structure stabilization, presenting a right-handed super-helix formed by four α-helices. Prolamin superfamily includes several protein families, but their identified aeroallergens are mainly included in the nsLTP and α-amylase families. Presently, this superfamily occupies the third place in terms of identified aeroallergens, totalizing 24 molecules [17].

#### nsLTP

The nsLTP constitute a large family of proteins that are abundant in all plants. Most nsLTP are extracellular proteins associated with cell walls, possessing lipid-binding specificity in their 3D structure. nsLTP are also known as the PR-14 family, whose members can intervene in the modulation of lipid composition, lipid transfer, vesicular trafficking, and signal transduction. nsLTP are small proteins with 9.5–10.5 kDa, presenting compact 3D structures [50], consisting of four α‐helices, which are stabilized by four highly conserved disulphide bridges and connected by flexible loops [51], forming an internal, tunnel‐like, hydrophobic cavity, responsible for the transport of various lipids. Allergic reactions triggered by inhalant nsLTP can range from mild to potentially life-threatening, such as anaphylaxis [52]. nsLTP are classified as panallergens due to their high homology and wide presence among plant species. Presently, 16 nsLTP have been identified as aeroallergens (e.g. Amb a 6, ragweed) (Fig. 1h) in pollens and leaves (Table 1) [17]. For several years, nsLTP-sensitized allergies were more prevalent in the Mediterranean region, but currently, this paradigm has changed with nsLTP progressively functioning as primary sensitizers in other geographical areas [51, 53, 54]. The nsLTP are related to important clinical conditions, such as LTP syndrome and pollen-food allergy syndrome (PFAS) [55].

#### α-Amylases

α-Amylases are metalloenzymes found in distinct glycoside hydrolase families, being ubiquitously distributed. They are present in different sources, namely microorganisms, plants and animals, displaying biological functions that regard the catalysis of α-1,4-glucosidic bonds in starch and related α-glucans with retention of α-anomeric configuration in the products [56]. The amylolytic digestion produces glucose, maltose, and maltodextrins. In addition, many α-amylases are also able to catalyze transglycosylation. Structurally, α-amylases encompass a single polypeptide chain folded into three domains (A, B and C). These enzymes present a characteristic (β/α)_8_-barrel with conserved catalytic core (domain A), a protrusion between the third strand and the third helix of (β/α)_8_-barrel having irregular β-like structure (domain B) and a C-terminal end of the amino acid sequence with key motif (domain C) (Table 1) [57].

Presently, 8 α-amylases have been identified as aeroallergens in mites (e.g., Der p 4, European HDM) (Fig. 1i), insects (e.g., Bla g 11, German cockroach), and fungi (e.g., Asp o 21, rice mold). α-Amylases have been described to cause occupational asthma due to the inhalation of flours and powders commonly present at bakeries, as well as in pharmaceutical and laboratory facilities [58, 59]. More recently, α-amylase from yellow mealworm has also been reported to be the culprit aeroallergen-inducing occupational allergy in workers contacting with mealworms in different circumstances, namely in pet stores, live fish bait or infested stored grains, as well as in mealworm farming for animal feed and human consumption [60].

### Subtilisin-like Serine Protease

Subtilisin-like proteases (subtilases) are a family of serine proteases exhibiting a characteristic catalytic triad consisting of aspartate, histidine, and serine [61]. These enzymes have a unique structural fold, comprising a highly twisted seven-stranded β-sheet flanked by two layers of α-helices. Subtilases are typically monomers although homodimerization can also occur [62]. Their MW varies from 34–58 kDa [17], depending on the location of the enzymes (vacuolar versus extracellular) (Table 1). Their physiological role depends on the allergen source, participating in the production of nutrients (through protein cleavage), protein turnover, fungi/plant defense system, and immune evasion [63]. Allergenic subtilases can alter the epithelial integrity through degradation of tight or adherens junctions, thus facilitating their penetration into submucosal tissues. Their contact with immune cells, facilitated by the epithelial barrier degradation, allows the cleavage of surface receptors on target cells, which optimizes the Th2-biased inflammatory response. Presently, it is the fourth family gathering more aeroallergens, totalizing 21 subtilases belonging to different sources, namely fungi (e.g., Cur l 4, mold) (Fig. 1j) and mites (e.g., Per a 10, American cockroach). Allergic symptoms associated with subtilisin sensitization may include breathlessness, sweating, and wheezing at exposures, contributing to occupational asthma [64].

### Expansin Superfamily

The expansin superfamily encompasses a set of secreted proteins (25–27 kDa) expressed in plants and divided into four families: α-expansin, β-expansin, expansin-like A, and expansin-like B. Current identified aeroallergenic β-expansins are included in the commonly known Grass group-1 allergens. Their biological role is related to cell wall loosening processes, thereby aiding in the pollination process [65]. The β-expansins are greatly preserved, sharing 60–70% of sequence identity among different grass species, thus contributing to their high potential cross-reactivity phenomenon [66, 67]. The β-expansins are structurally divided into two domains: (i) the N-terminal domain 1, which is similar to the catalytic domain of family 45 glycoside hydrolases (GH45); and (ii) domain 2 that entails β-sheets with 36% sequence identity to group 2 and 3 grass pollen allergens (Table 1) [65].

Presently, there are 14 β-expansins (e.g., Zea m 1, maize) (Fig. 1k) identified as important plant aeroallergens. They are known to cause IgE-reactivity in more than 90% of grass pollen-allergic patients [68], being considered major pollen allergens based on their high prevalence and potency, especially in the Poaceae family. Additionally, two expansin-like proteins have also been identified as aeroallergens, namely Sor h 2 (Johnson grass) and Phl p 3 (timothy), belonging to group 2 and 3 grass pollen allergens, respectively, containing a C-terminal expansin domain. Like the β-expansins, these expansin-like proteins are also considered major allergens, revealing IgE-reactivity with more than 50% of the human sera tested [69, 70].

### Ole e 1-like Proteins

The Ole e 1-like protein family can originate exclusively from various plant pollens [71]. The Ole e 1-like proteins usually range from 15–20 kDa, with Ole e 1, the main allergen of this family, displaying a MW of around 16 kDa [17]. Ole e 1-like protein family encompasses a seven-strand β-barrel fold stabilized by three conserved disulfide bonds and the [EQT]-G-x-V–Y-C-D-[TNP]-C-R consensus pattern [71, 72]. The biological role of these proteins is yet to be fully described, although they are reported to be mostly related to plant fertilization (Table 1) [73]. In fact, protein content is increased right before the plant initiates pollen tube emergence. Pollen grains conserve the metabolic mechanism at the dehydrated stage to reserve an important quantity of Ole e 1-like proteins to aid in pollen tube growth [73].

Fra e 1 from ash pollen also constitutes the major allergen of the source and contributes mainly to spring pollinosis [74]. Ole e 1-like proteins exhibit a high degree (> 82%) of sequence identity among Oleaceae members [71]. Presently, 16 Ole e 1-like proteins are predominantly identified as aeroallergens in pollens (e.g., Lig v 1, privet) (Fig. 1l) and fungi (e.g., Asp f 35, common mold). These allergens are well-known for causing pollen-related symptoms such as asthma, seasonal allergic rhinitis, and oral allergy syndrome.

### PR-10 Protein (Bet v 1) Family

The PR-10 proteins represent a significant group of allergens found in birch pollen-related allergies, also known as Bet v 1 family of proteins. PR-10 proteins typically have a MW of 15–17 kDa, exhibit slight acidity and resistance to proteases, being structurally different from the other PR proteins. Although presenting low sequence identity, PR-10 proteins share a highly conserved structure featuring a curved, seven-stranded antiparallel β-sheet enveloping a 25-amino acid α-helix (α3) at the C-terminus. Additionally, two short α-helices (α1 and α2) positioned between the β1 and β2 strands complete the framework, forming a hydrophobic core that serves as a ligand-binding site. Functionally, PR-10 proteins are used for plant defense mechanisms, providing answers to biotic/abiotic stresses, and transporting amphiphilic compounds (fatty acids, cytokines, flavonoids, and sterols) across cellular barriers (Table 1) [75].

In Northern-European countries, the PR-10 protein family comprise the prime triggers of PFAS, with European white birch pollen Bet v 1 (Fig. 1m) triggering primary sensitization [76]. Bet v 1 is the best-characterized PR-10 member and it is the major causative agent of seasonal allergies in Central Europe and North America [77], being considered a major allergen of RA. In the referred regions, more than 95% of patients are allergic to birch pollen, with almost 60% being exclusively sensitized to Bet v 1 [78].

Birch pollen allergy is the leading cause of winter and spring pollinosis in the temperate climate zones of the Northern Hemisphere, affecting over 100 million allergic individuals sensitized to Bet v 1 [79]. This aeroallergen is characterized as a highly promiscuous ligand acceptor, being potentially responsible for its strong allergenicity. Bet v 1 allergen has unique structural properties, which may contribute to its high allergenic potential. Additionally, birch pollen extracts have been found to possess proteolytic activity, which can degrade tight junction proteins [79]. Clinically, the significance of PR-10 proteins lies in their association with a wide array of symptoms, ranging from mild to potentially life-threatening, especially when correlated with FA [80]. Currently, twelve PR-10 proteins are identified as aeroallergens in pollen, being the common cause of respiratory allergies, like asthma or allergic rhinitis.

### Tropomyosins

Tropomyosin is ubiquitously expressed in animals and fungi, serving as a universal regulator of the actin cytoskeleton. Tropomyosins are composed of ~ 284 residues (MW of 34–38 kDa), forming coiled-coil homo- or heterodimeric structures that polymerize along the length of actin. Each tropomyosin molecule consists of two parallel α-helices featuring two sets of 7 alternating actin-binding sites, being only biologically active when in dimers (Table 1) [81]. Known primarily for regulating the actin-myosin interaction during muscle contraction, tropomyosins are also fundamental for the organization and dynamics of the cytoskeleton [82]. Tropomyosins are essential contractile elements, presenting highly conserved sequences and structures across vertebrates and invertebrates.

Tropomyosins are only allergenic in invertebrates, being important food allergens in various shellfish species, but they are also minor aeroallergens in arthropods (HDM and cockroaches), where they can constitute up to 1% of muscle mass [83, 84]. So far, 11 tropomyosins have been identified as aeroallergens in insects/mites (e.g., Der p 10, European HDM) (Fig. 1n) [17].

Tropomyosin was the first identified allergen implicated in the cross-reactivity among dust mites, crustaceans, and insects, with > 80% of the crustacean-sensitized patients reacting to tropomyosin and exhibiting severe allergic symptoms. Tropomyosins evidence exceptionally high sequence identity among crustaceans (95–100%) and a great degree of sequence homology (75–83%) with HDM and insect tropomyosins [85]. Tropomyosin Der p 10 shares > 65% of identical residues with other invertebrate tropomyosins [86] and its sensitization prevalence varies between 9–18%, in Europe [87]. Half of the European patients allergic to HDM and sensitized to Der p 10 are at risk of experiencing clinically relevant cross-reactivity when consuming seafood [88] or their vapors during cooking.

## Conclusions

Since allergenic proteins have been recognized as important biological/immunological triggers, their identification, characterization, and systematization have been attempted. Their inclusion into protein families sharing physicochemical properties aims at facilitating their study, while predicting their likelihood to be identified as potential allergens. Therefore, user-friendly databases, efficiently compiling data on allergens, are much needed. Presently, few databases with relevant information are freely available for consultation, but they frequently lack updates. In this Part 1 review, it was also verified that most works on aeroallergen characterization/identification were published in the 1990’s or early beginning of 2000’s, whose data were rarely refined or re-analyzed with new analytical methodologies. This suggests that key data on the immunological/chemical characterization of some aeroallergens are still scarce. This fact highlights the need to revise and confirm the data generated with obsolete analytical tools in order to provide clear answers, namely in the inclusion of allergens into different protein families.

In summary, the inclusion of aeroallergens in their correct protein families should facilitate the prediction of the physicochemical properties linked to protein allergenicity. Evaluating the impact of such features might provide some valuable insights in predicting the allergenicity of novel inhalant proteins, which justifies the need of extending this review to Part 2.

## Data Availability

No datasets were generated or analysed during the current study.
